# Cutaneous metastasis in endometrial cancer: once in a blue moon – case report

**DOI:** 10.1186/1477-7819-12-86

**Published:** 2014-04-04

**Authors:** David Atallah, Nadine el Kassis, Fouad Lutfallah, Joelle Safi, Charbel Salameh, Samah Nadiri, Lina Bejjani

**Affiliations:** 1 Department of Gynecology and Obstetrics, Hôtel-Dieu de France University Hospital, Saint Joseph University, Bvd. Alfred Naccache, Achrafieh, P.O. Box: 116-5137, Museum, Beirut, Lebanon; 2 Department of Anatomy-Pathology, Hôtel-Dieu de France University Hospital, Saint Joseph University, Bvd. Alfred Naccache, Achrafieh, P.O. Box: 116-5137, Museum, Beirut, Lebanon

**Keywords:** Bleeding, Cutaneous metastasis, Endometrial carcinoma, Lesions

## Abstract

**Background:**

Cutaneous metastases from internal malignancies are uncommon. Moreover, endometrial carcinoma rarely metastasizes to the skin, with a reported prevalence of 0.8%. Here, we report the case of a 62-year-old woman who developed cutaneous metastases from an endometrial carcinoma.

**Case presentation:**

When admitted to our department, the patient underwent a biopsy that showed the presence of cutaneous metastasis in relation to her initial endometrial cancer, diagnosed 3 years earlier. Thereafter, she was treated with a bilateral uterine artery embolization and chemotherapy. The patient had complications and survived 5 months after the diagnosis of the cutaneous metastasis. She died from sepsis.

**Conclusion:**

Cutaneous metastases of the endometrial carcinoma are usually incurable and suggest an unfortunate prognosis where palliation is the mainstay of patient management.

## Background

Cutaneous metastases may occur as the initial manifestation of internal malignancy or late in the course of the disease. The incidence of cutaneous metastases in internal malignancies has been reported between 0.7% and 10% [1]. Incidence of various tumors metastasizing to the skin correlates with the sex-wise frequency of occurrence of various primary malignancies. As such, lung cancer (1.7 to 3.1%) and breast cancer (23.9%) are the commonest epithelial malignancies metastasizing to the skin in men and women respectively [2-4]. Furthermore, although endometrial carcinoma is the most common gynecological cancer, it rarely metastasizes to the skin, with a reported prevalence of 0.8% [5].

In this paper, we report the case of a 62-year-old woman with cutaneous metastasis of the endometrial cancer and describe the clinical and pathological features. We also discuss the clinical and histopathological criteria for the differential diagnosis of cutaneous paraneoplastic lesions and cutaneous metastases. Moreover, we review the literature to determine and to evaluate both the prognostic significance and the frequency of this disease.

## Case presentation

**Figure 1:**
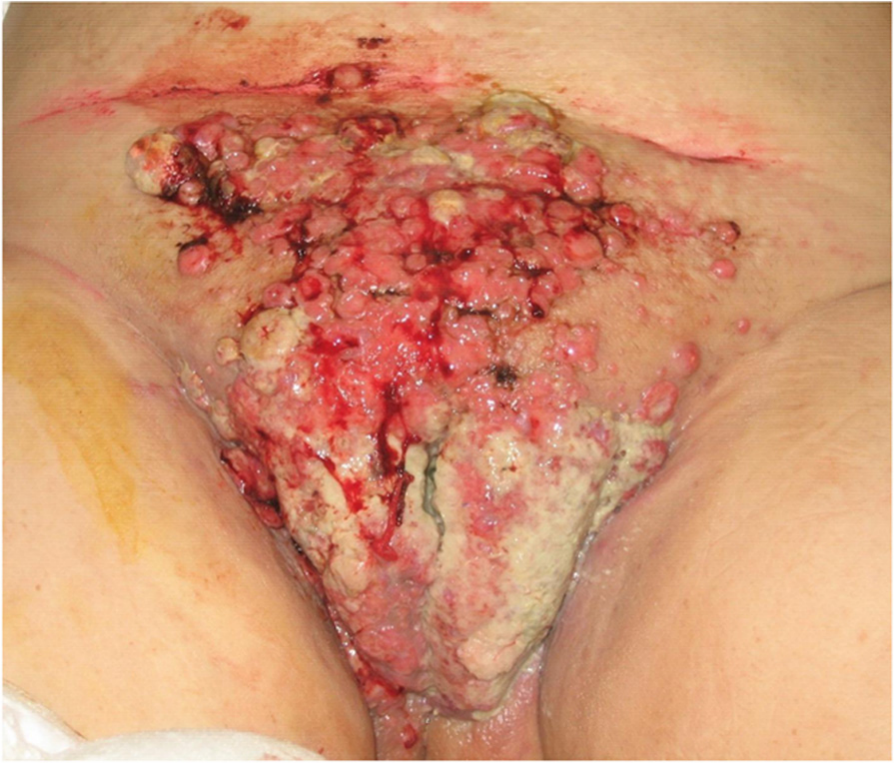
Cutaneous metastasis of the endometrial carcinoma.

A 62-year-old female, Gravida 2, Para 2, was referred to our department for itching with identifiable pelvic cutaneous lesions. She was menopausal at age 51 years. Clinical examination revealed the presence of cutaneous papulo-bullous lesions located at the site of the laparotomy incision extending down to the vulva and up to the umbilicus (Figure 1). No pelvic mass was palpated on physical examination. A thoraco-abdomino-pelvic computed tomography scan was performed and showed no abnormality.

The patient’s previous gynecological history revealed that she was diagnosed 3 years earlier with endometrial carcinoma. The histological type of the endometrial adenocarcinoma was endometrioid and defined as Féderation Internationale de Gynécologie et d’Obstétrique stage 1B, grade 2. A total hysterectomy, bilateral salpingo-oophorectomy, and pelvic lymph node dissection were performed. Pelvic lymph nodes were negative (seven nodes). The patient did not undergo any para-aortic lymphadenectomy. External pelvic radiotherapy was prescribed, leading to a complete clinical response. During the last 3 years, the patient had regular check-ups with her oncologist outside our institution.

**Figure 2:**
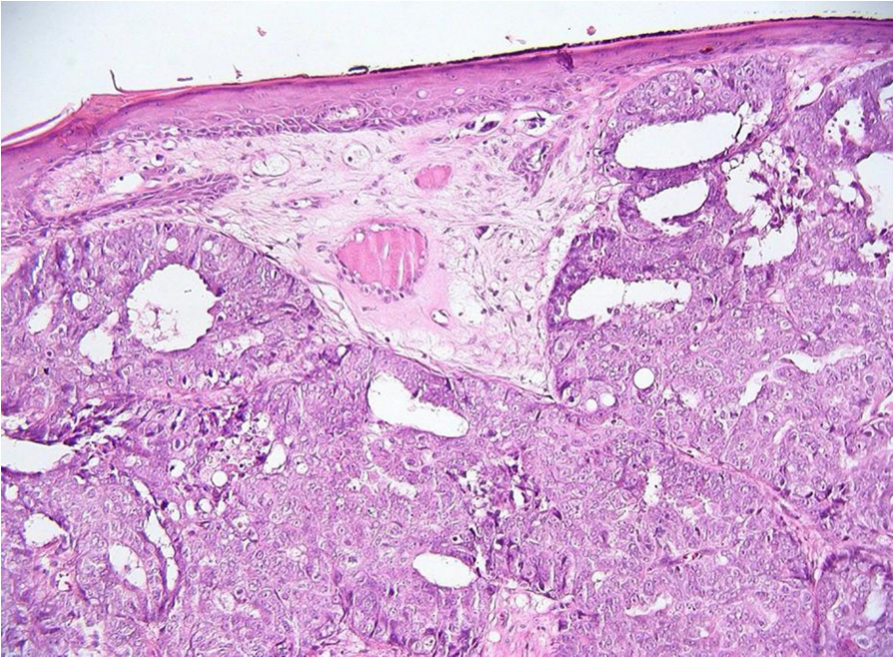
**Histopathological findings of the skin nodule.** Medium-power photomicrograph (original magnification, ×200; hematoxylin and eosin stain) of the skin nodule, showing a moderately differentiated endometrioid adenocarcinoma.

When admitted to our department, the patient underwent a biopsy that showed the presence of cutaneous metastasis in relation to her initial cancer (Figure 2). Thereafter, she had a bilateral uterine artery embolization because of important vaginal bleeding, and four cycles of salvage chemotherapy with etoposide (VP-16) and cisplatin [6-8].

Three months later, the patient was transfused with 3 units of packed red blood cells because of recurrent massive vaginal bleeding. She then underwent pelvic magnetic resonance imaging, which revealed the presence of a pelvic tumor mass of 15 cm × 8 cm × 4 cm invading the bladder and rectum. However, pelvic computed tomography angiography identified no signs of active arterial bleeding. The last months of the patient were marked by episodes of urinary tract and scar incision infections (extended-spectrum beta-lactamase *Escherichia coli* and *Pseudomonas aeruginosa*) treated with antibiotics. The patient died 2 months later from sepsis.

## Conclusions

Distinguishing between cutaneous paraneoplastic lesions and those due to invasion of the skin through hematogeneous spread, lymphatic spread or by direct extension from a primary tumor is essential. Paraneoplastic dermatoses follow the evolution of the underlying cancer and are due to the production by the tumor of hormones, cytokines and growth factors [9].

Histopathologically, the type of endometrial carcinoma that disseminated to the skin was endometrioid adenocarcinoma in our case – as the majority of studies that analyze skin metastases from endometrial cancer also report [10]. A mixed Müllerian tumor has also been reported. In these lesions, neoplastic cells appear to be arranged in a gland-like pattern within the dermis with different grades of atypia. Metastatic skin disease from uterine papillary serous carcinoma, an aggressive variant of endometrial carcinoma mimicking serous papillary ovarian carcinoma, has been documented [10]. Clinically, cutaneous metastasis can take any form of lesions including nodules, papules, ulcers, plaques and tumors, with usually four histopathological forms involving the dermis; namely, nodular, infiltrative, diffuse and intravascular [11]. These lesions may be the only manifestation of an underlying visceral cancer.

The frequency of cutaneous metastasis is correlated to the frequency of each malignancy, which is why women with cutaneous metastases most frequently have the following primary malignancies: breast, ovary, oral cavity, lung and large intestine [10]. Globally, cutaneous metastases represent 2% of all cutaneous neoplasms [12].

Although endometrial carcinoma is the most common gynecological cancer, it only rarely metastasizes to the skin, with a reported prevalence of 0.8% [5]. A systematic English-language literature search on PubMed between 1966 and 2013 using the terms ‘endometrial carcinoma’, ‘skin’, ‘cutaneous’, ‘metastasis’ and ‘spread’ identified only 26 cases of cutaneous metastasis in endometrial cancer [13-19]. Most of these reports emphasize the rarity of this pattern of dissemination. Four points illustrated in the current case deserve to be discussed.

The strategy for cancer treatment and management in cutaneous metastases is to determine the tumor origin, which is achievable with tissue biopsy of the metastatic nodule. However, this may prove unhelpful in settings where patients would have visited several hospitals and had surgeries without histological diagnosis or confirmation of excised lesions [14]. Contrarily, in our case, biopsy enabled us to confirm the presence of cutaneous metastasis in relation to the initial endometrial cancer of the patient.

Secondly, in the reported cases, metastasis from endometrial cancer has been most commonly noted at the site of initial surgery [16], such as in our patient. The initial surgical and radiotherapy site must therefore be examined carefully for skin metastasis. More rarely, distant cutaneous sites, including scalp, toes and trunk, have been reported [20,21].

Thirdly, cutaneous metastases may vary in numberfrom a single nodule to greater than 20 lesions, similarly to our case.

Finally, cutaneous metastases of the endometrial carcinoma are associated with poor prognosis and a mean life expectancy reported as approximately 4 to 12 months after the diagnosis of cutaneous metastasis [22,23]. The factor influencing survival is the time elapsed between diagnosis and the appearance of the skin recurrences [24]. Furthermore, to date there are no treatments with proven efficacy because of the rarity of this specific site of metastasis. The treatment for most patients is palliative, and although chemotherapy and radiotherapy are often used in these patients, they are ineffective in many cases [25]. In our case, the patient underwent embolization and etoposide and cisplatin chemotherapy. Etoposide and cisplatin regimen was prescribed given that these two agents are more efficacious than the carboplatin and paclitaxel regimen in the treatment of advanced endometrial cancer, as reported by Olawaiye and colleagues in 2012 [6]. Kim and colleagues [7] and Pierga and colleagues [8] also demonstrated that the combination of etoposide and cisplatin is an effective regimen with an acceptable toxicity in patients with recurrent or metastatic endometrial carcinoma. Moreover, we considered that the patient had a second-line treatment for metastatic endometrial carcinoma, although the standard of care for women who present with locally advanced or who experience relapse with metastatic disease is to proceed with an anthracycline, taxane and platinum combination [26]. However, the disease evolution was rapidly fatal and the patient died 5 months after her admission to our department due to sepsis.

In conclusion, both our case and the other cases reported in the literature demonstrate that the appearance of a cutaneous lesion at the site of initial surgery a few years or months after the diagnosis of endometrial carcinoma could represent widespread dissemination of the primary tumor. These cutaneous metastases herald a poor prognosis and fatal evolution.

## Consent

Consent for publication for the patient was sought from the next of kin of the patient. A copy of the written consent is available for review by the Editor-in-Chief of this journal to be published.

## Competing interests

The authors declare that they have no competing interests.

## Authors’ contributions

DA participated in the treatment and follow-up of the patient, and wrote the article. SN and DA participated in the histopathological diagnosis. NeK, FL, JS, CS and LB participated in the writing of article. All authors read and approved the final manuscript.
